# Robot-Assisted Boari-flap after Orthotopic Neobladder Using the KangDuo Surgical Robot-01 System

**DOI:** 10.1590/S1677-5538.IBJU.2025.0341

**Published:** 2025-08-30

**Authors:** Guanpeng Han, Xiang Wang, Zhihua Li, Kunlin Yang, Liqun Zhou, Xuesong Li

**Affiliations:** 1 Peking University First Hospital Department of Urology Beijing China Department of Urology, Peking University First Hospital, Beijing, China; 2 Peking University Institution of Urology Beijing China Institution of Urology, Peking University, Beijing, China; 3 National Urological Cancer Center Beijing China National Urological Cancer Center, Beijing, China

## Abstract

**Purpose:**

Benign ureteroenteric anastomosis stricture (BUES) is a well-recognized long-term complication following urinary diversion ( 1 ). While endourological interventions are often first-line, their success rates are limited ( 2 , 3 ). Open uretero-ileal reimplantation remains the gold standard but is technically challenging and carries high complication risks ( 2 ). Robotic surgery offers a promising alternative with comparable success rates and minimally invasive benefits ( 4 ). In addition to the da Vinci® system, several new surgical robotic systems have been developed, demonstrating comparable safety and efficacy ( 5 - 7 ). This study reports our experience with robotic-assisted Boari-flap using the KangDuo-Surgical-Robot-01 (KD-SR-01) System in managing long-segment BUES after radical cystectomy with orthotopic neobladder.

**Materials and Methods:**

A 64-year-old man developed left BUES 2.5 years after robot-assisted radical cystectomy with orthotopic neobladder. After nephrostomy drainage for 6 months, a robotic-assisted Boari-flap was performed using the KD-SR-01 system in the Trendelenburg position. Surgical steps included: neobladder mobilization, distal ureter dissection, neobladder flap creation, ureter-flap anastomosis, and flap tubularization.

**Results:**

Surgery was successful without conversion. The stricture length was 5 cm. The neobladder flap measured 5 × 3 cm (length × width). Operating time was 145 minutes, with 30 mL of blood loss. The nephrostomy tube and double-J stent were removed two months postoperatively. At three-month follow-up, the patient remained asymptomatic with stable serum creatinine. Cine magnetic resonance urography demonstrated normal ureteral peristalsis and ureteral jets. No postoperative complications occurred.

**Conclusions:**

Robotic-assisted Boari-flap after radical cystectomy with orthotopic neobladder is technically feasible. A larger cohort with longer follow-up is necessary to assess its safety and effectiveness.

**Available at:** VIDEO

## Data Availability

Not applicable
